# Cerebrospinal Fluid Inflammatory Cytokine Aberrations in Alzheimer's Disease, Parkinson's Disease and Amyotrophic Lateral Sclerosis: A Systematic Review and Meta-Analysis

**DOI:** 10.3389/fimmu.2018.02122

**Published:** 2018-09-19

**Authors:** Xi Chen, Yang Hu, Zongze Cao, Qingshan Liu, Yong Cheng

**Affiliations:** Key Laboratory of Ethnomedicine for Ministry of Education, Center on Translational Neuroscience, College of Life and Environmental Sciences, Minzu University of China, Beijing, China

**Keywords:** inflammation, cytokine, neurodegenerative diseases, cerebrospinal fluid, meta-analysis

## Abstract

It has been suggested that cytokine-mediated inflammation plays a key role for the onset and/or development of neurodegenerative diseases including Alzheimer's disease (AD), Parkinson's disease (PD) and Amyotrophic lateral sclerosis (ALS). However, clinical studies have yielded inconsistent results for the aberrant cytokine levels in circulation of patients with AD, PD, and ALS. Previous studies have used meta-analysis to address the inconsistent data for blood cytokine levels in the patients with AD, PD, and ALS. Here, we performed a systemic review of cerebrospinal fluid inflammatory cytokine data in patients with AD, PD and ALS, and sought to quantitatively summarize the CSF inflammatory cytokine data with a meta-analytical technique. The systematic search from Pubmed and Web of Science identified 71 articles with 2629 patients and 2049 controls for the meta-analysis. Random-effects meta-analysis demonstrated that CSF TGF-β, MCP-1, and YKL-40 levels were significantly elevated in AD patients when compared with controls. In addition, patients with PD had heightened levels of TGF-β1, IL-6, and IL-1β in CSF. Furthermore, G-CSF, IL-2, IL-15, IL-17, MCP-1, MIP-1α, TNF-α, and VEGF levels were significantly increased in patients with ALS as compared with controls. Taken together, these results not only strengthen the clinical evidence that neurodegenerative diseases are accompanied by the increased inflammatory response, but also reveal the unique inflammatory response profile in the central nervous system of patients with AD, PD and ALS. Given the robust associations between some cytokines and neurodegenerative diseases found in this meta-analysis, CSF inflammatory cytokines may be used as biomarkers for these diseases in the future.

## Introduction

Neurodegenerative diseases such as Alzheimer's disease (AD), Parkinson's disease (PD), and Amyotrophic lateral sclerosis (ALS) are devastating diseases affect millions of people globally (1–3). Some of the patients suffered from AD, PD, or ALS might benefit from a few drugs for symptom relief,but there are no treatments to stop or slow down the progress of these diseases (4–10). Although the pathologies have been well characterized for these neurodegenerative diseases, the etiologies of AD, PD, and ALS are far from being understood. Therefore, there is urgent need to better understand the etiology of AD, PD and ALS, and subsequently develop new effective therapies for these neurological disorders.

In the last decade, increasing evidence suggest that inflammation is associated with the onset and development of the neurodegenerative diseases including AD, PD, and ALS (11–15). These are also evidenced by the post-mortem studies showing increased microglia cell activation in the brain of the patients with AD, PD or ALS (16–18). Moreover, increased circulating inflammatory cytokine releases have been found to be associated with the pathogenesis of these neurodegenerative disease (19–21). Although clinical data were inconsistent for individual cytokines and across studies, a meta-analysis published in 2010 included 40 studies and demonstrated that patients with AD had elevated peripheral blood levels for tumor necrosis factor (TNF)-alpha, interleukin (IL)-1β, IL-6, IL-12, IL-18, and transforming growth factor (TGF)-β, whereas blood IL-4, IL-8 and IL-10 did not show significant differences between AD patients and controls (22). Our recent meta-analyses showed significant associations between peripheral blood IL-6, tumor necrosis factor, IL-1β, IL-2, IL-10, C-reactive protein, and RANTES levels and PD (23), and significant higher blood levels of TNF-α, TNFR1, IL-1β, IL-6, IL-8, and VEGF levels in ALS patients when compared with controls (24). Therefore, these meta-analyses clarified the peripheral blood cytokine levels in AD, PD and ALS with relatively large sample size, and provided potential biomarkers for these neurodegenerative diseases.

In addition to the significant associations between peripheral blood cytokine levels and the neurodegenerative diseases, studies have also found aberrant cytokine levels in the cerebrospinal fluid (CSF) of patients with AD, PD and ALS (19, 20, 25, 26). The measurements of the CSF inflammatory cytokine levels in these neurodegenerative diseases have generated great interest in recent years, given that they have the potential to better explain the etiology of the diseases. However, the clinical studies have yielded inconsistent results for CSF cytokine levels in AD, PD and ALS patients. Some studies suggested increased CSF inflammatory cytokine levels in patients with AD, PD and ALS (27–29), whereas other studies did not show significance differences between cases and controls for CSF cytokine concentrations (30–32), and a few studies even showed decreased inflammatory cytokines in CSF of patients with these diseases (33, 34). Therefore, to address the inconsistent clinical data on CSF cytokine in AD, PD, and ALS patients, a systematic review with meta-analysis is warranted.

## Method

This systematic review and meta-analysis followed the guidelines that are recommended by the PRISMA statement (Preferred Reporting Items for Systematic reviews and Meta-Analysis) (35).

### Search strategy and study selection

**Figure 1:**
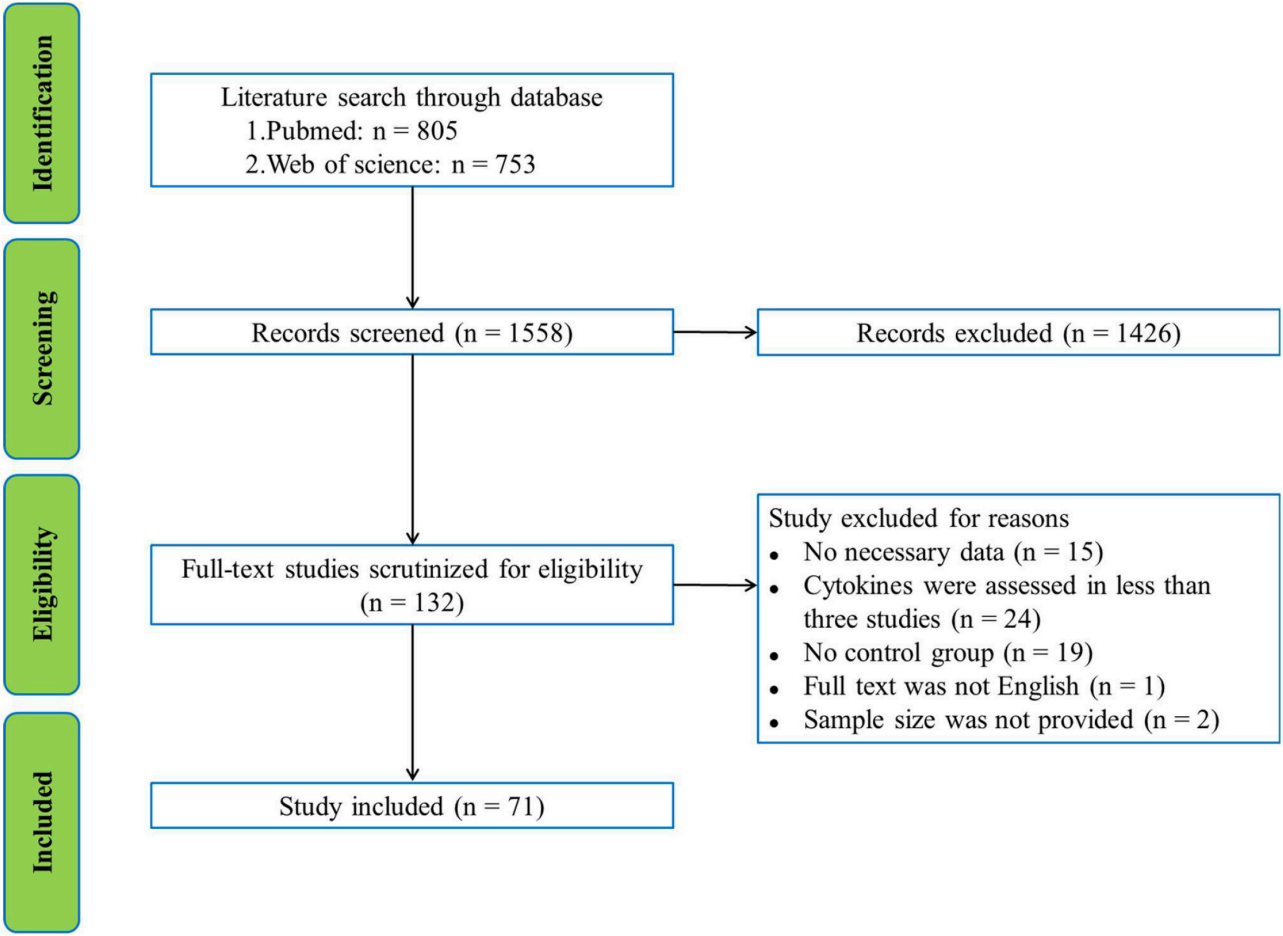
PRISMA flowchart of the literature search.

Pubmed and Web of science were used to search English language publications by two independent investigators through March 2018. The database search used the following search terms: (inflammation or cytokine or chemokine or tumor necrosis factor or interleukin or interferon or C-reactive protein) and (Alzheimer's disease or amyotrophic lateral sclerosis or Parkinson's disease) and (CSF or cerebrospinal fluid), without year restriction. The initial search yielded 805 records from Pubmed and 753 records from Web of Science with the search term. After screening the titles and abstracts, 1426 records were excluded because they were not related to our present subject. The remaining 132 articles were selected for full-text scrutiny. 61 studies were excluded due to: no necessary data (*n* = 15); cytokines were assessed in less than three studies (*n* = 24), no control group (*n* = 19); full text was not English (*n* = 1); sample size was not provided (*n* = 2). Therefore, a total of 71 studies with 2629 patients and 2049 controls were included in this meta-analysis (Supplementary Material). A flowchart of the selection process was presented in Figure 1.

### Data extraction

Sample size, *P*-value, mean cytokine concentrations and standard deviation (s.d.) were extracted from all the articles included in this meta-analysis as the main data. Additional data such as average age of patients, percentage of male patients, dementia severity (Mini-Mental State Examination, MMSE), medication status and assay type were also extracted (Supplementary Table 1).

### Statistical analysis

The Comprehensive Meta-Analysis Version 2 software (Biostat, Englewood, NJ, USA) was applied for all the statistical analyses. Effective sizes (ESs) were mainly generated by sample size, mean cytokine concentration and s.d, and the rest ESs were calculated by sample size and *P*-value if the mean cytokine concentration and s.d. we're not reported. Standardized mean differences in cytokine levels between groups were calculated as ESs, which can be converted to Hedge's g that provides unbiased ESs adjusted for sample sizes (36). The statistical difference of the pooled ES was estimated by the 95% confidence interval (95% CI). We hypothesized that within-study and between-study moderators would influence the true ES, therefore the random-effects model was chosen for this meta-analysis. Sensitivity analysis was undertaken by removing one study at a time to test if the outcomes of the meta-analysis were significantly influenced by a single study.

Cochran Q test was applied to evaluate statistical difference of heterogeneity across studies. It was considered statistically significant when *P*-value < 0.1. I^2^ index was used to determine the inconsistency across studies which evaluated the impact of heterogeneity, and we used 0.25, 0.50, and 0.75 of I^2^ to indicate small, medium and high levels of heterogeneity (37). Unrestricted maximum-likelihood random-effects meta-regressions of ESs were used to evaluated if the mean age, gender (proportion of male), MMSE, disease duration and publication year were served as confounders to affect the ESs. The Egger's test was used to determine the significance of a statistical test for publication bias, which assesses the degree of funnel plot asymmetry (38).

If not specified, *P*-value < 0.05 was considered statistically significant in this meta-analysis.

## Results

### Main association of AD, PD, ALS with cytokine levels

**Figure 2:**
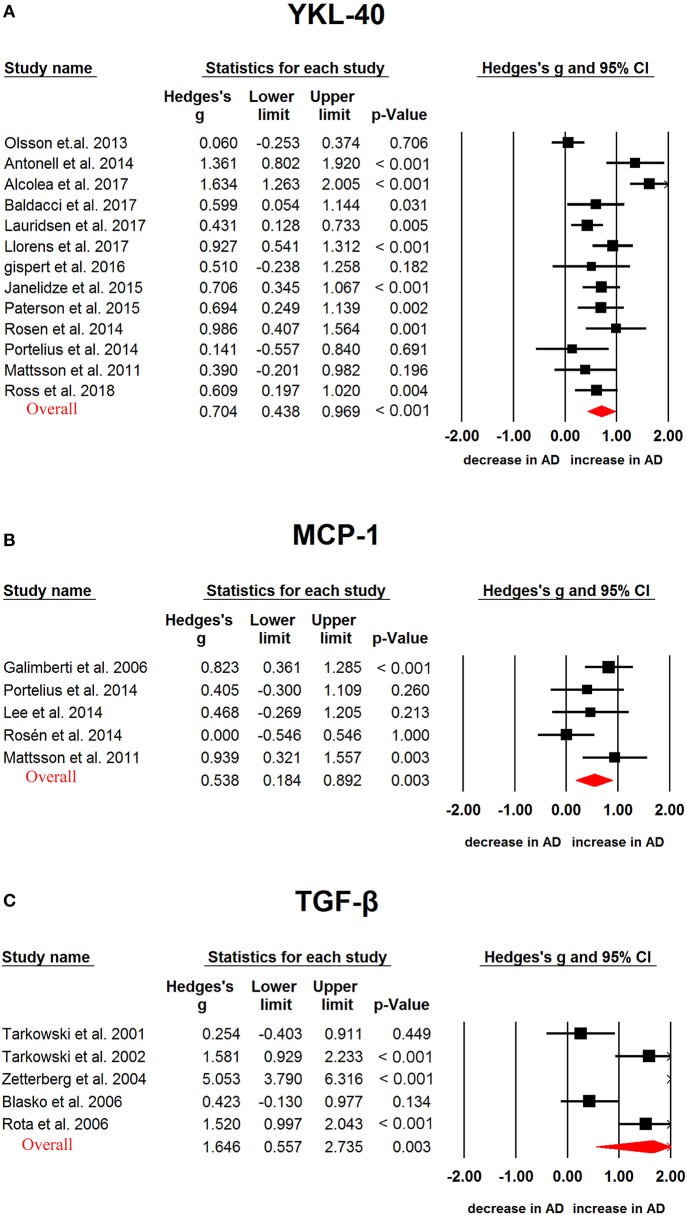
Studies of cerebrospinal fluid YKL-40, MCP-1 and TGF-β in Alzheimer's disease. Forest plot displaying random-effects meta-analysis results of the association between YKL-40 **(A)**, MCP-1 **(B)**, TGF-β **(C)** and Alzheimer's disease. The sizes of the squares are proportional to study weights.

We used random-effects meta-analysis and compared the CSF cytokine levels between AD, PD, ALS and controls. CSF TGF-β (Hedges g, 1.646; 95% CI, 0.557-2.735; *P* = 0.003), MCP-1(Hedges'g, 0.538; 95% CI, 0.184-0.892; *P* = 0.003) and YKL-40 (Hedges'g, 0.704; 95% CI, 0.438-0.969; *P* < 0.001) levels were significantly increased in AD patients as compared with controls (Figure 2, Table 1). In contrast, CSF IL-1β, IL-6, IL-8, and TNF-α levels did not significantly associate with AD (Table 1).

**Table 1:** Summary of Comparative Outcomes for Measurements of CSF Cytokine Levels.

**Disease**	**Cytokine**	**No.of studies**	**No.With disease/ controls**	**Main effect**			**Heterogeneity**				**Publication bias**	
				**Hedges g (95% CI)**	**z score**	***P*-value**	**Q statistic**	**df**	***P*-value**	***I*^2^ statistic**	**Egger intercept**	***P*-value**
**AD**	IL-1β	6	128/121	−0.084 (−0.526 to 0.358)	−0.372	0.710	14.162	5	0.015	64.695	−2.725	0.379
	IL-6	12	305/281	0.093 (−0.203 to 0.390)	0.617	0.537	33.120	11	0.001	66.787	−1.333	0.522
	IL-8	4	92/79	0.290 (−0.228 to 0.808)	1.098	0.272	7.853	3	0.049	61.800	−5.124	0.156
	MCP-1	5	114/117	0.538 (0.184 to 0.892)	2.975	0.003	6.979	4	0.137	42.685	−1.430	0.735
	TGF-β	5	126/119	1.646 (0.557 to 2.735)	2.962	0.003	54.011	4	< 0.001	92.594	9.728	0.152
	TNF-α	5	137/90	0.325 (0.007 to 0.643)	−1.670	0.095	40.076	4	< 0.001	90.019	−6.024	0.363
	YKL-40	13	700/555	0.704 (0.438 to 0.969)	5.190	< 0.001	55.050	12	< 0.001	78.202	0.937	0.690
**PD**	IL-6	6	237/151	0.468 (0.049 to 0.887)	2.191	0.028	18.585	5	0.002	73.096	6.140	0.025
	IL-1β	4	140/98	0.370 (0.033 to 0.707)	2.154	0.031	4.200	3	0.241	28.575	0.262	0.940
	TGF-β1	3	91/75	0.472 (0.147 to 0.798)	2.842	0.004	0.383	2	0.826	0	1.786	0.837
	TNF-α	3	142/89	0.826 (−0.027 to 1.678)	1.898	0.058	15.849	2	< 0.001	87.381	7.910	0.194
**ALS**	G-CSF	5	251/166	0.552 (0.345 to 0.759)	5.218	< 0.001	4.227	4	0.376	5.365	2.336	0.270
	GM-CSF	4	214/133	0.317 (0.119 to 0.515)	1.557	0.119	11.454	3	0.010	73.808	1.519	0.762
	IFNγ	7	312/210	1.231 (−0.103 to 2.565)	1.808	0.071	240.522	6	< 0.001	97.505	12.421	0.299
	IL-2	5	251/166	0.317 (0.119–0.515)	2.063	0.039	9.506	4	0.050	57.919	1.471	0.677
	IL-4	5	166/130	0.068 (−0.161 to 0.297)	0.584	0.559	2.343	4	0.673	0	3.357	0.053
	IL-5	4	146/110	0.265 (−0.172 to 0.703)	1.188	0.235	8.775	3	0.032	65.812	1.030	0.883
	IL-6	10	311/241	0.153 (−0.174 to 0.480)	0.917	0.359	29.760	9	< 0.001	69.758	−1.680	0.442
	IL-7	5	166/130	0.254 (−0.076 to 0.584)	1.510	0.131	7.820	4	0.098	48.852	2.955	0.475
	IL-8	7	195/163	0.311 (−0.052 to 0.674)	1.681	0.093	16.785	6	0.010	64.255	−0.238	0.946
	IL-10	7	280/199	−0.064 (−0.583 to 0.455)	−0.242	0.809	42.029	6	< 0.001	85.724	1.328	0.735
	IL-12p70	4	146/110	0.221 (−0.070 to 0.512)	1.488	0.137	4.011	3	0.260	25.208	2.777	0.518
	IL-13	4	146/110	0.062 (−0.185 to 0.309)	0.495	0.621	1.860	3	0.602	0	3.884	0.003
	IL-15	4	120/121	0.4594 (0.032 to 0.886)	2.108	0.035	7.154	3	0.067	58.063	1.878	0.733
	IL-17	5	151/116	0.743 (0.494 to 0.993)	5.842	< 0.001	2.580	4	0.630	0	0.427	0.863
	MCP-1	18	478/356	0.593 (0.321 to 0.866)	4.367	< 0.001	51.988	17	< 0.001	67.300	−0.867	0.423
	MIP-1α	6	292/198	0.902 (0.096 to 1.707)	2.194	0.028	78.592	5	< 0.001	93.638	−8.696	0.368
	MIP-1β	7	291/205	0.628 (−0.233 to 1.489)	1.430	0.513	114.115	6	< 0.001	94.742	−11.485	0.305
	RANTES	5	149/111	0.291 (−0.213 to 0.795)	1.131	0.258	15.367	4	0.004	73.970	1.592	0.765
	TNF-α	6	175/143	0.355 (0.039 to 0.672)	2.202	0.028	9.377	5	0.095	46.680	3.250	0.212
	VEGF	9	365/254	0.507 (−0.001 to 1.014)	1.956	0.050	68.377	8	< 0.001	88.300	−8.439	0.043

*AD, Alzheimer's Disease; ALS, Amyotrophic lateral sclerosis; df, degrees of freedom; G-CSF, Granulocyte colony-stimulating factor; GM-CSF, Granulocyte-macrophage colony-stimulating factor; IFN-γ, interferon γ; IL, interleukin; MCP, Monocyte Chemoattractant Protein; MIP, Macrophage Inflammatory Proteins; PD, Parkinson disease; RANTES, regulated on activation, normal T-expressed, and presumably secreted; TNF, tumor necrosis factor; TGF, transforming growth factor; VEGF, Vascular endothelial growth factor; YKL-40, Chitinase-3-like protein 1*.

**Figure 3:**
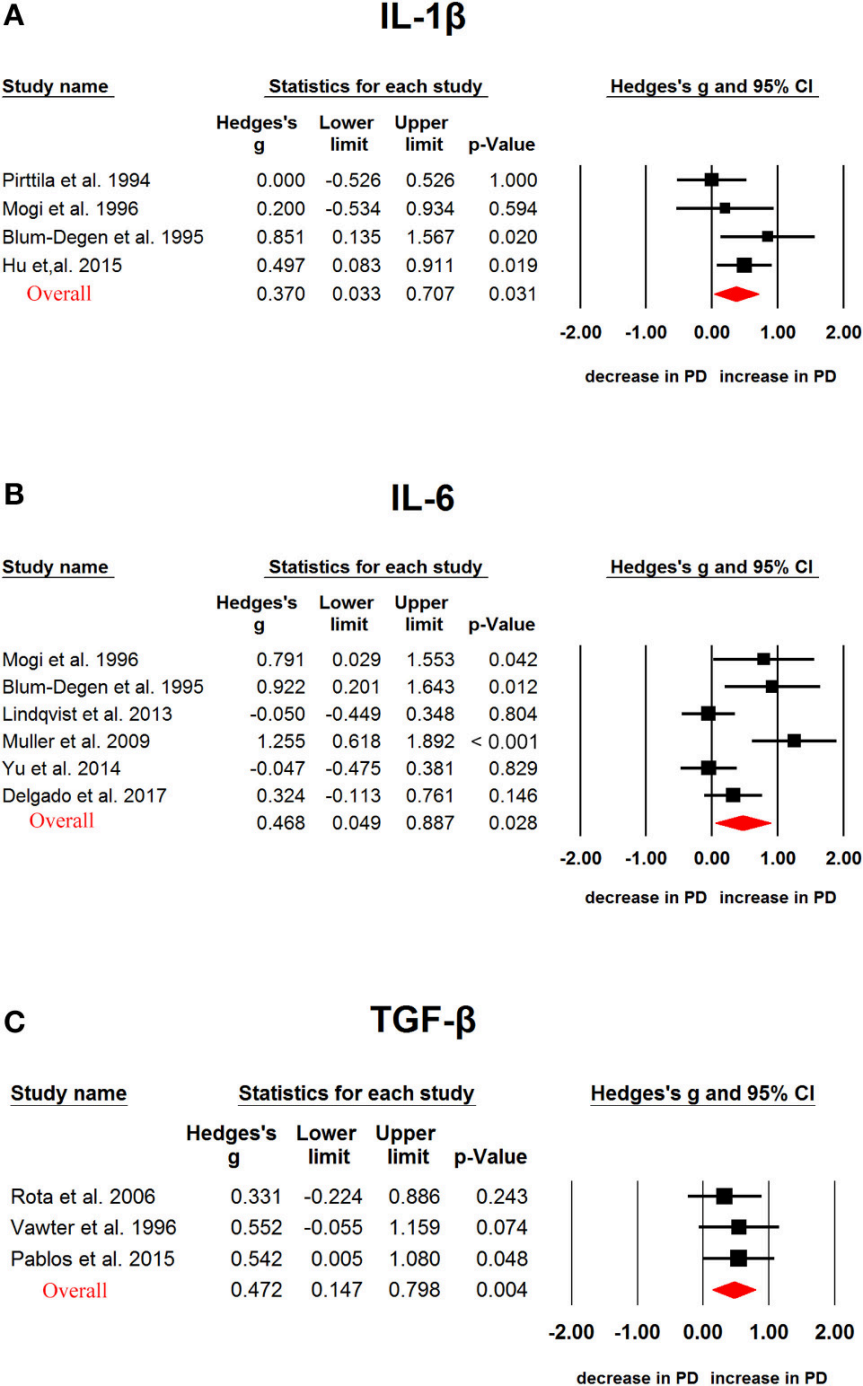
Studies of cerebrospinal fluid IL-1β, IL-6 and TGF-β1 in Parkinson's disease. Forest plot displaying random-effects meta-analysis results of the association between IL-1β **(A)**, IL-6 **(B)**, TGF-β1 **(C)** and Parkinson's disease. The sizes of the squares are proportional to study weights.

We next analyzed CSF cytokine levels in PD patients, and the results showed that PD patients had significantly higher CSF TGF-β1 (Hedges'g, 0.472; 95% CI, 0.147-0.798; *P* = 0.004), IL-6 (Hedges'g, 0.468; 95% CI, 0.049-0.887; *P* = 0.026) and IL-1β (Hedges'g, 0.370; 95% CI, 0.033-0.707; *P* = 0.031) levels than controls (Figure 3, Table 1), whereas CSF TNF-α (Hedges'g, 0.826; 95 % CI,−0.027-1.678; *P* = 0.058) levels had no significant ES estimate (Table 1).

**Figure 4:**
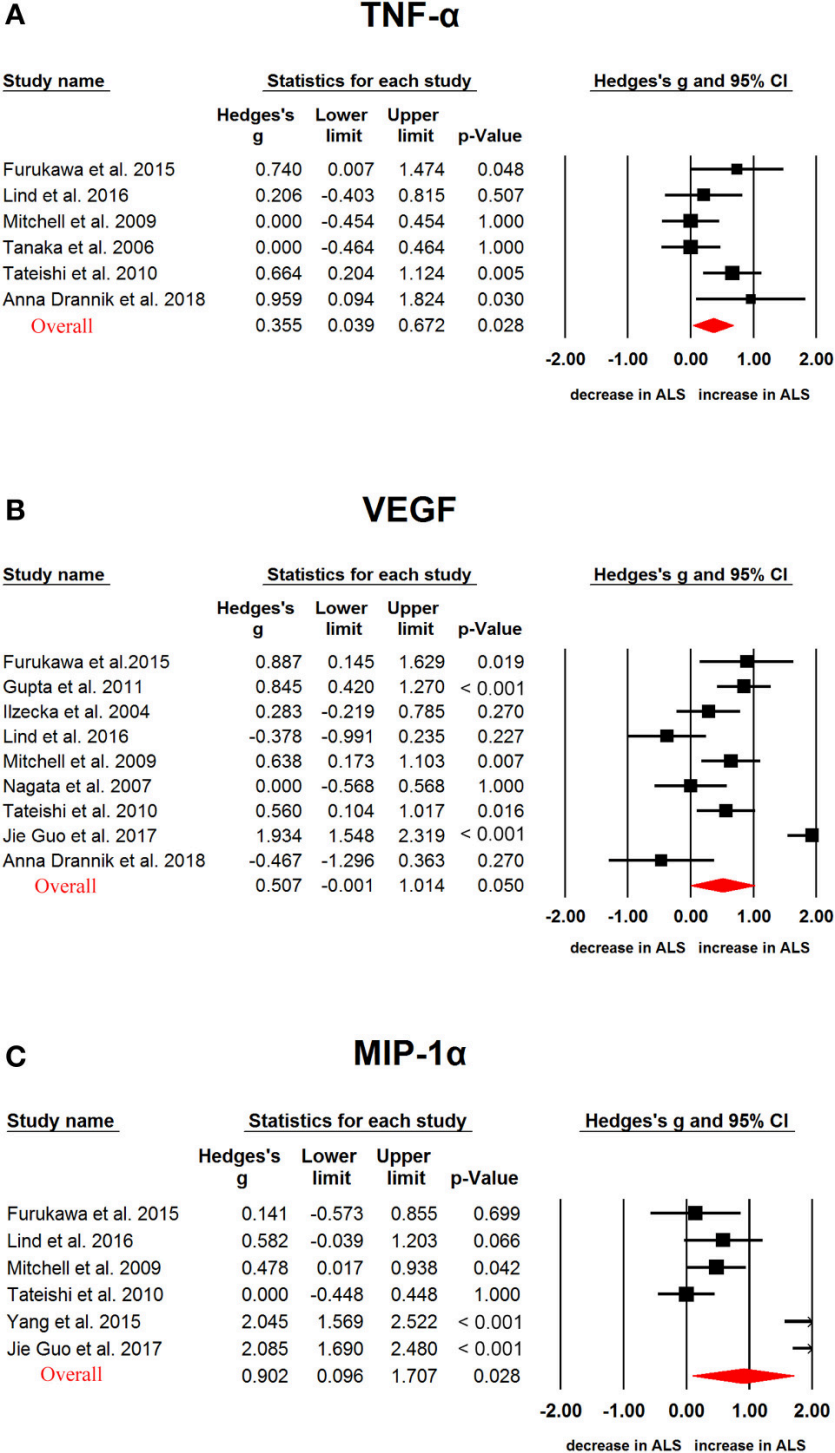
Studies of cerebrospinal fluid TNF-α, VEGF and MIP-1α in Amyotrophic lateral sclerosis. Forest plot displaying random-effects meta-analysis results of the association between TNF-α **(A)**, VEGF **(B)**, MIP-1α **(C)** and Amyotrophic lateral sclerosis. The sizes of the squares are proportional to study weights.

**Figure 5:**
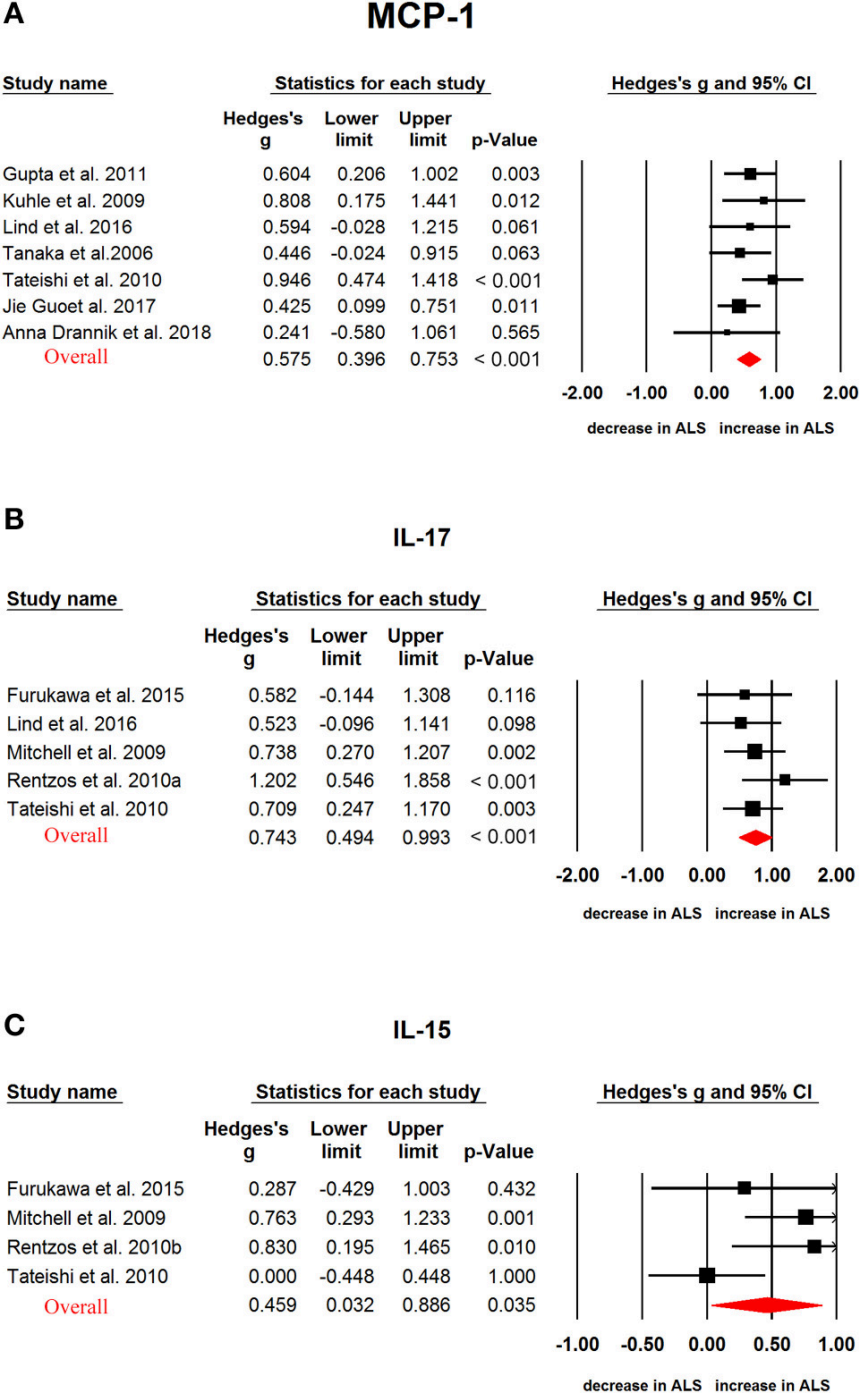
Studies of cerebrospinal fluid MCP-1, IL-17, and IL-15 in Amyotrophic lateral sclerosis. Forest plot displaying random-effects meta-analysis results of the association between MCP-1 **(A)**, IL-17 **(B)**, IL-15 **(C)** and Amyotrophic lateral sclerosis. The sizes of the squares are proportional to study weights.

**Figure 6:**
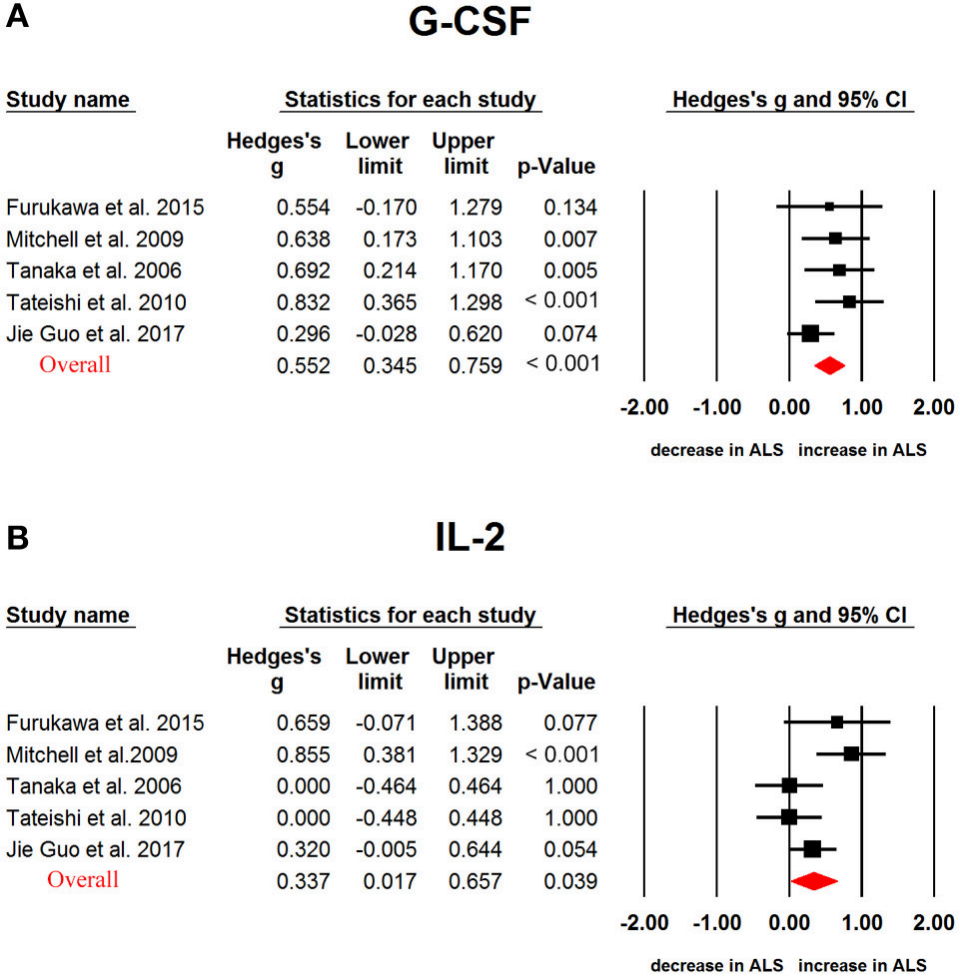
Studies of cerebrospinal fluid G-CSF and IL-2 in Amyotrophic lateral sclerosis. Forest plot displaying random-effects meta-analysis results of the association between G-CSF **(A)**, IL-2 **(B)** and Amyotrophic lateral sclerosis. The sizes of the squares are proportional to study weights.

In ALS, G-CSF (Hedges'g, 0.552; 95% CI, 0.345-0.759; *P* < 0.001), IL-2 (Hedges'g, 0.317; 95% CI, 0.119-0.515; *P* = 0.002), IL-15 (Hedges'g, 0.4594; 95% CI, 0.032-0.886; *P* = 0.035), IL-17 (Hedges'g, 0.743; 95% CI, 0.494-0.993; *P* < 0.001), MCP-1 (Hedges'g, 0.593; 95% CI, 0.321-0.866; *P* < 0.001), MIP-1α (Hedges'g, 0.902; 95% CI, 0.096-1.707; *P* = 0.028), TNF-α (Hedges'g, 0.355; 95% CI, 0.039-0.672; *P* = 0.028) and VEGF (Hedges'g, 0.507; 95% CI,−0.001-1.014; *P* = 0.050) levels were significantly increased in the patients when compared with controls (Figures 4, 5 and 6, Table 1). However, GM-CSF, IFNγ, IL-4, IL-5, IL-6, IL-7, IL-8, IL-10, IL-12p70, IL-13, MIP-1β, RANTES levels did not show significant differences between cases and controls (Table 1).

### Investigation of heterogeneity

In AD, 6 of 7 cytokines had significant heterogeneity among studies. TGF-β, TNF-α, and YKL-40 presented high levels of heterogeneity, IL-6, IL-8 and IL-1β presented moderate levels of heterogeneity, and MCP-1 showed small levels of heterogeneity (Table 1). In PD, TNF-α, IL-6 and IL-1β showed high, moderate, small levels of heterogeneity, respectively, whereas TGF-β1 did not show heterogeneity among studies (Table 1). In ALS, G-CSF, IL-4, IL-13, and IL-17 did not present between-study heterogeneity, whereas the heterogeneity ranged from small to large for other cytokines included in this meta-analysis (Table 1).

We next analyzed potential moderators that may explain the heterogeneity in the meta-analysis. We considered assay type and control type as potential categorical variables, and age, sex, disease duration, disease severity and publication year as potential continuous variables. For those cytokines significantly associated with AD, PD and ALS, only YKL-40 levels in AD and MCP-1 levels in ALS were assessed in more than ten studies. We therefore attempted to perform subgroup and/or meta-regression analyses for YKL-40 in AD, and MCP-1 in ALS.

The subgroup analysis stratified by control type (healthy control group [HC] and disease control group [DC]) suggested that CSF MCP-1 levels were increased in ALS patients as compared to HC subjects (4 studies, Hedges' g = 0.520, 95% CI = 0.297 to 0.743, *P* < 0.001), or DC subjects(15 studies, Hedges' g = 0.607, 95% CI = 0.254 to 0.960, *P* = 0.001), and no heterogeneity was found for the HC group (Q = 1.724, d.f. = 3, I^2^ = 0.000, *P* = 0.632), whereas the impact of heterogeneity was slightly increased for the DC group (Q = 48.772, d.f. = 14, I^2^ = 71.295, *P* < 0.001). Furthermore, the impact of heterogeneity was slightly increased (Q = 49.25, d.f. = 13, I^2^ = 73.605, *P* < 0.001), and the significant association between CSF MCP-1 and ALS was retained for the ELISA method (14 studies, Hedges' g = 0.528, 95% CI = 0.161 to 0.895, *P* = 0.005). For the non-ELISA method, there was no significant heterogeneity among studies (Q = 2.393, d.f. = 3, I^2^ = 0, *P* = 0.495), and the significant association between CSF MCP-1 and ALS was retained (4 studies, Hedges' g = 0.696, 95% CI = 0.40 to 0.962, *P* < 0.001). Interestingly, the impact of heterogeneity was reduced to zero (Q = 2.53, d.f. = 5, I^2^ = 0, *P* = 0.772), and the significant association between CSF MCP-1 and ALS was slightly increased for the ES generated by sample size and *P*-value group (6 studies, Hedges' g = 0.685, 95% CI = 0.479 to 0.891, *P* < 0.001). However, the significant association between CSF MCP-1 and ALS was lost for the ES generated by mean concentration, s.d. and sample size group (12 studies, Hedges' g = 0.459, 95% CI = −0.011 to 0.93, *P* = 0.056), and the heterogeneity increased approximately 10% (Q = 48.921, d.f. = 11, I^2^ = 77.515, *P* < 0.001). These results suggest that assay type, ES generation method and control type may partially explain the between-study heterogeneity in this meta-analysis.

We then performed meta-regression analyses and demonstrated that sex, age, disease severity, and publication year had no moderating effects on the significant association between CSF YKL-40 and AD (*p* > 0.05 in all the analyses). Similarly, age, sex, disease duration and publication year did not confound the outcome of the meta-analysis for the association between CSF MCP-1 and ALS (*p* > 0.05 in all the analyses).

Sensitivity analysis demonstrated that no single study could influence the statistically significant differences in CSF YKL-40 levels between AD patients and controls, and CSF MCP-1 levels between ALS patients and controls. In addition, no significant risk for publication bias was observed for the included studies analyzing CSF YKL-40 in AD and MCP-1 in ALS, as demonstrated by the Egger's test (Table 1).

## Discussion

To the best of our knowledge, this meta-analysis is the first work to pool data from studies evaluating CSF inflammatory cytokine levels in various neurodegenerative diseases as compared with controls. Our analyses suggested significant increases of CSF inflammatory cytokine levels for TGF-β, MCP-1, and YKL-40 in patients with AD when compared with controls. In addition, the CSF TGF-β1, IL-6, and IL-1β levels were significantly increased in patients with PD when compared with controls. More CSF cytokines including G-CSF, IL-2, IL-15, IL-17, MCP-1, MIP-1α, TNF-α, and VEGF were significantly associated with ALS. Although previous studies presented conflicting results for the inflammatory cytokine releases in the central nervous system (CNS) of neurodegenerative diseases, this meta-analysis provided strong clinical evidence with large sample size to validate increased CNS inflammatory response in AD, PD, and ALS.

The level of CSF MCP-1 increased both in patients with AD and ALS, suggesting this chemokine may represent a common inflammatory pathway to affect the two diseases. The same explanation could apply to the increased CSF TGF-β levels in AD and PD. The other cytokines included in this meta-analysis did not show significant associations with at least two neurodegenerative diseases, indicating the unique signature of the CNS inflammatory response of individual neurodegenerative disease. However, the non-significant associations between some CSF cytokines and AD, PD, or ALS may due to the limited number of studies with small sample size, making the observation of significant associations difficult. For example, our results showed significant increase of CSF TNF-α concentration in patients with ALS, but a non-significant association between CSF TNF-α levels and AD (*P* = 0.095) or PD (*p* = 0.058), this phenomenon is likely because of the small number of studies included in the meta-analysis, and the high levels of heterogeneity among studies. We hypothesize with increasing number of studies and larger sample size, CSF TNF-α levels would significantly associate with AD and PD. Therefore, future studies are necessary to evaluate whether heightened central TNF-α releases occur in all the three neurodegenerative diseases analyzed in the meta-analysis, and thereby better explain the etiology of AD, PD and ALS. Nevertheless, these results suggested shared and unique CNS inflammatory responses in different neurodegenerative diseases.

Previous meta-analyses have addressed the inconsistent results for blood inflammatory cytokine releases in AD (22), PD (23), and ALS (24). However, it was unclear whether peripheral levels of cytokines reflect their levels in CNS. Although the meta-analysis by Swardfager et al. published in 2010 included AD cytokine data both in CSF and blood (22), the limited number of studies with small sample size only demonstrated significantly increased TGF-β levels in the CSF of AD patients. Moreover, the inconsistent data for CSF cytokines levels in patients with PD and ALS found in various clinical studies (28, 32) further confounded relationship between the peripheral and central levels of inflammatory cytokines. In this study, we included studies published after 2010, and demonstrated increased CSF TGF-β, MCP-1, and YLK-40 level in patients with AD. The CSF data is consistent with the significantly increased blood TGF-β concentrations in AD (22). However, blood IL-1β and IL-6 showed significant associations with AD, but not CSF IL-1β and IL-6. In PD, the results from our study and the previous meta-analysis (23) revealed consistent alterations of IL-1β and IL-6 both in blood and CSF. Furthermore, the consistent changes of cytokine TNF-α and VEGF in peripheral and central of ALS patients are supported by our previous (24) and present meta-analyses. In contrast, the abnormal expression profile of other cytokines in the two movement disorders were not consistent in peripheral and central. These results suggest that at least some cytokine dysregulations in peripheral reflect the changes of cytokines in the CNS of patients with neurodegenerative diseases, these cytokines in peripheral therefore have the potential to be indicators for what occur in the brains of patients with AD, PD or ALS.

The between-study heterogeneity ranged from zero to high. The advantage of this study is that a meta-analytic technique with subgroup and meta-regression analyses was used to adjust for potential confounders. Although meta-regression analyses did not find any continuous variables that may have moderating effect on the outcome for the meta-analysis, the subgroup analyses suggested that different control type (DC vs HC) and assay type (ELISA vs non-ELISA) partially explained the heterogeneity. However, the small heterogeneity in some subgroups may be due to the limited number of studies, given that data were pooled from four studies comparing CSF MCP-1 levels between ALS patients and HC subjects, and four studies used non-ELISA assay to compare CSF MCP-1 levels between ALS patients and controls. It should be noted that out of the four studies, two studies used bead-based fluorescent method to detect MCP-1 levels. Therefore, another possibility of the low between-study heterogeneity for the non-ELISA method was due to the relatively new technology with potential more consistent results, although future studies are necessary to validate this idea. Furthermore, another interesting finding is the zero between-study heterogeneity for the sub-group generating ES from sample size and *P*-value. This phenomenon could be due to the relatively small number of studies (6 studies) for the sub-group analyzing CSF MCP-1 levels in ALS patients. However, the second explanation is that studies with positive results are more likely to report only sample size and *P*-value.

Although this meta-analysis provided strong clinical evidence to clarify the CSF cytokine aberrations in AD, PD and ALS, there are several limitations in this study. The first limitation is that most cytokines in AD, PD, or ALS were studied in less than 10 studies, thus it is unclear whether the non-significant associations between some cytokines and neurodegenerative diseases are due to the limited number of studies with small sample size, and it also prevented us from analyzing the potential confounding factors that explained the between-study heterogeneity. The second limitation is that some of the detailed information of clinical variables, these include disease duration, disease severity and medication status, were not reported in the original papers, and therefore precluded us from assessing the effects of these potential moderators on the outcomes of the meta-analysis. Therefore, more clinical studies are needed to address the inflammatory responses in the CNS of AD, PD, and ALS with more controls on the clinical variables, thereby to better understand the etiology of the neurodegenerative diseases. The third limitation of this study is that we only included English-language publications, and this may potentially limit the number of studies in this meta-analysis. However, considering the very small number of non-English publications on cytokine levels in neurodegenerative diseases, this is unlikely to significantly affect the outcomes of the meta-analysis.

## Author contributions

YC and QL conceived and designed the study, XC and YH collected the data. XC, YH, ZC, QL, and YC analyzed and interpreted the data. YC drafted the manuscript with critial revisons from all the authors.

### Conflict of interest statement

The authors declare that the research was conducted in the absence of any commercial or financial relationships that could be construed as a potential conflict of interest.
