# Supplementary material for: Cerebrospinal Fluid Inflammatory Cytokine Aberrations in Alzheimer's Disease, Parkinson's Disease and Amyotrophic Lateral Sclerosis: A Systematic Review and Meta-Analysis
Source: Front Immunol. 2018 Sep 19;9:2122. doi: 10.3389/fimmu.2018.02122 (PMC6156158; doi:10.3389/fimmu.2018.02122)
Supplement: Supplementary file 1 [file Table_1.DOCX]

**Supplementary Table. Characteristics of included studies measuring CSF cytokine concentrations**

| **Study/Year** | **Cytokines Measured** | **Country** | **Samples**  **(Disease/Ctrl)** | **Gender (%Male) (Disease/Ctrl)** | **Mean Age** **(Disease/Ctrl)** | **Mean Disease duration** | **MMSE** | **Diagnosis** | **Assay type** |
| --- | --- | --- | --- | --- | --- | --- | --- | --- | --- |
| Olsson et al. 2013 | YKL-40 | Sweden | AD(96)/HC(65) | 35.4/26.2 | 76.2/74.7 | / | 19.0 | NINCDS-ADRDA | ELISA |
| Antonell et al. 2014 | YKL-40 | Spain | AD(22)/HC(43) | 40.9/27.9 | 68.4/61.6 | / | 24.9 | NA | ELISA |
| Alcolea et al. 2017 | YKL-40 | Spain | AD(72)/HC(76) | 38.9/40.8 | 70.8/60.2 | / | 21.6 | NA | ELISA |
| Baldacci et al. 2017 | YKL-40 | Italy | AD(35)/HC(21) | 31.4/38.1 | 73.0 /64.0 | / | 23.0 | NA | ELISA |
| Lauridsen et al. 2017 | YKL-40 | Italy | AD(66)/HC(41) | 41.9/45.0 | 61.0/68.0 | / | 29.0 | NINCDS-ADRDA | ELISA |
| Llorens et al. 2017 | YKL-40 | Spain | AD(65)/HC(50) | 33.8/46.0 | 67.0 /70.0 | / | N | NINDS-AIREN | ELISA |
| Galimberti et al. 2006 | IP-10, MCP-1, IL-8 | Italy | AD(36)/DC(41) | 36.1/36.6 | 64.0/64.0 | / | 16.9 | NINCDS-ADRDA | rate nephelometry. |
| Gispert et al. 2017 | YKL_40 | Spain | AD(8)/HC(44) | 12.5/36.0 | 65.9/ 62.2 | / | 23.0 | NA | ELISA |
| Janelidze et al. 2015 | YKL-40 | Sweden | AD(74)/HC(53) | 32.0/30.0 | 75.3/76.4 | / | 19.4 | NINCDS-ADRDA | ELISA |
| Paterson et al. 2015 | YKL-40 | UK | AD(61)/HC(30) | 26.2/46.7 | 62.5 /59.8 | / | 20.6 | NA | ELISA |
| Rosén et al. 2014 | MCP-1, YKL-40, | UK | AD(25)/HC(25) | 36.0/36.0 | 67.2 /60.6 | / | 21.0 | NINCDS-ADRDA | ELISA |
| Portelius et al. 2014 | CCL2, YKL-40 | USA | AD(12)/HC(20) | 50.0/67.0 | 41.0/40.0 | / | N | NA | ELISA |
| Correa et al. 2011 | CCL2 | Brazil | AD(22)/HC(27) | 22.7/55.5 | 74.7/64.4 | / | NA | NINCDS-ADRDA | ELISA |
| Lee et al. 2014 | IL-1β,IL-6,IL-8, TNF-α,MCP-1 | USA | AD(16)/HC(12) | NA | N | / | N | NA | ELISA |
| Llano et al. 2011 | IL-1β,IL-6,IL-8, TNF-a | Urbana | AD(15)/HC(7) | 80.0/71.4 | 70.2/65.0 | / | 22.1 | NINCDS-ADRDA | MULTI-SPOT Human Cytokine Assay |
| Mattsson et al. 2011 | CCL2，YKL-40，IL6，IL8 | Sweden | AD(25)/HC(19) | 44.0/47.4 | 76/75 | / | N | NINCDS-ADRDA | ELISA |
| Wennström et al. 2015 | IL-6 | Sweden | AD(45)/HC(36) | 28.9/44.4 | 76.0 /62 .0 | / | 20.0 | NINCDS-ADRDA | ELISA |
| Yamada et al. 1995 | IL-6 | Japan | AD(12)/HC(7) | 25.0/71.3 | 73.0/72.0 | / | NA | NINCDS-ADRDA | ELISA |
| Hampel et al. 1999 | IL-6 | Germany | AD(25)/HC(19) | 44.0/57.9 | 63.0/66.0 | / | 21.7 | NINCDS-ADRDA | ELISA |
| Tarkowski E et al. 1999 | IL-1β, IL-6, TNF-α | Sweden | AD(34)/HC(25) | 41.2/NA | 63.0/65.0 | / | NA | NA | ELISA |
| Martinez et al. 2000 | IL-1β, IL-6 | Spain | AD(10)/HC(10) | 40.0/50.0 | 70.0/67.0 | / | 17.5 | NINCDS ADRDA; DSM III-R | NA |
| Tarkowski et al. 2000 | TNF-α | Sweden | AD(52)/HC(25) | 44.2/NA | 74.0/68.0 | / | 20.7 | NINCDS-ADRDA | ELISA |
| Rösler et al. 2001 | IL-6 | Austria | AD(27)/HC(49) | 33.3/54.2 | 69.0/61.0 | / | 14.3 | NINCDS-ADRDA | ELISA |
| Tarkowski E et al. 2001 | IL-4, TGF-β | Sweden | AD(20)/HC(15) | 40.0/NA | 63.0/65.0 | / | NA | NINCDS-ADRDA | ELISA |
| Tarkowski E et al. 2002 | TGF-β | Sweden | AD(20)/HC(17) | 40.0/NA | 63.0/NA | / | NA | NINCDS-ADRDA | ELISA |
| Gomez-Tortosa et al. 2003 | IL-1β, IL-6 | Spain | AD(33)/HC(46) | 30.3/52.2 | 74.0/73.0 | / | 16.8 | NINCDS-ADRDA | ELISA |
| Zetterberg et al. 2004 | TGF-β | Sweden | AD(20)/HC(20) | 30.0/30.0 | 77.0/65.0 | / | NA | NINCDS-ADRDA | NA |
| Richartz E et al. 2005 | IL-1β, IL-6, TNF-α | Germany | AD(20)/HC(21) | 20.0/66.7 | 72.0/68.0 | / | 16.0 | NINCDS-ADRDA | ELISA |
| Blasko et al. 2006 | TGF-β | Austria | AD(23)/HC(27) | 34.8/48.1 | 72.0/67.0 | / | 20.7 | NINCDS-ADRDA | ELISA |
| Rota et al. 2006 | TGF-β | Italy | AD(30)/HC(25) | 40.0/60.0 | 71.0/69.0 | / | 16.0 | NINCDS ADRDA; DSM-IV | ELISA |
| Galimberti et al. 2008 | IL-6 | Italy | AD(43)/HC(30) | 27.9/33.3 | 66.0/63.0 | / | 22.1 | NINCDS-ADRDA | ELISA |
| Paterson et al. 2018 | YKL-40 | UK | AD(114)/HC(29) | 42.3/46.7 | 62.5/63.5 | / | 22.0 | NINCDS-ADRDA | ELISA |
| Pirttila et al. 1994 | IL-1β | Finland | PD(20)/HC(42) | 50.0/38.1 | 64.6 /60.9 | NA | / | NA | ELISA |
| Mogi et al. 1996 | IL-1β,IL-6 | Japan | PD(14)/HC(13) | 50.0/76.9 | 68.0/46.0 | NA | / | NA | ELISA |
| Blum-Degen et al. 1995 | IL-6 | Germany | PD(22)/HC(12) | 54.6/50.0 | 61.0/61.0 | 0.5-3years | / | H & Y scale | ELISA |
| Rota et al. 2006 | TGF-β1 | Italy | PD(24)/HC(25) | 62.5/60.0 | 68.0/69.0 | 4.8 | / | H & Y scale | ELISA |
| Mogi et al. 1994 | TNF-α | Japan | PD(15)/HC(16) | 40.0/37.5 | 58.0/46.0 | NA | / | NA | EIA |
| Janelidze et al. 2015 | IL-8 | Sweden | PD(100)/HC(38) | 65.0/42.1 | 65.9/65.0 | 8.3 | / | H & Y scale | ELISA |
| J.Zhang et al. 2008 | IL-8 | US | PD(40)/HC(95) | 72.0/46.0 | 59.0/63.0 | NA | / | H & Y scale | ELISA |
| Lindqvist et al. 2013 | IL-6,TNF-α | Sweden | PD(87)/HC(33) | 64.37/42 | 65.6/65.8 | 8.1 | / | H & Y scale,UPDRS,Schwab & England scale | ELISA |
| T.Muller et al. 2009 | IL-6 | Germany | PD(22)/HC(22) | NA | 61.0/NA | NA | / | NA | ELISA |
| MartinPablos et al. 2015 | TGFβ1 | Spain | PD(37)/HC(21) | NA | NA | NA | / | NA | ELISA |
| Vawter et al. 1996 | TGFβ1 | US | PD(30)/HC(16) | 73.33/68.75 | 73.8/72.7 | NA | / | NA | ELISA |
| Hu et al. 2015 | IL-1β, TNF-α | China | PD(84)/HC(31) | NA | NA | NA | / | NA | ELISA |
| Yu et al. 2014 | IL-6,INF-γ | China | PD(62)/HC(31) | 53.33/54.8 | 59.6/52.2 | median 2.58 year | / | H & Y scale | ELISA |
| Delgado-Alvarado et al. 2017 | IL-6, TNF-a | Spain | PD(40)/HC(40) | 71.79/52.63 | 71.3/68.1 | NA | / | NA | ELISA |
| Baron et al. 2005 | MCP-1 | Italy | ALS(27)/ DC(30) | 48.1/40.0 | 55.6/48.5 | 19.4 | / | El Escorial | ELISA |
| Ford et al. 2004 | IL-6 | Australia | ALS (11)/DC(15) | 54.5/33.3/46.7 | 64.0/48.5/56.1 | NA | / | El Escorial | ELISA |
| Furukawa et al. 2015 | IL-1b, IL2, IL4, IL-5, IL6, IL-7, IL-8, IL-10, IL12(p70), IL-13, IL15, IL-17, G-CSF, GM-CSF, IFN-r, MCP-1, CCL3, CCL4, CCL5, TNFa, VEGF, | Japan | ALS(26)/DC(10) | 57.7/90.0 | 65.0/70.3 | 14.5±10.9 | / | El Escorial | Bio-Plex Pro Human Cytokine 27-plex Assay/ ELISA |
| Gupta et al. 2011 | VEGF-A, MCP-1 | India | ALS(50)/HC(50) | 76.0/78.0 | 47.4/40.0 | 19.0 | / | El Escorial | ELISA/ QuantiGlochemiluminescent assay |
| Gupta et al. 2012 | MCP-1 | India | ALS(44)/ HC(29) | 77.3/79.3 | 47.2/38.0 | 19.2 | / | El Escorial | ELISA |
| Iłzecka et al. 2004 | VEGF | Poland | ALS(30)/DC(30) | 60.0/46.7 | 55.0/53.0 | 26.0 | / | El Escorial | ELISA |
| Krieger et al. 1992 | IL-6 | Canada | ALS(15) /DC(20) | NA | NA | NA | / | NA | NA |
| Kuhle et al. 2009 | IL-8, MCP-1, MIP-1b (CCL4), | Switzerland | ALS(20)/DC(20) | NA | NA | days 281±132 | / | El Escorial | Sandwich immunoassay |
| Lehnert et al. 2014 | MCP-1, | Germany/Portugal/UK/Poland/Italy/Belgium | ALS(30)/DC(30) | see table 1 in the passage | see table 1 in the passage | NA | / | NA | ELISA |
| Lind et al. 2016 | CCL5, MCP-1, CCL-3, , IL17, VEGF, TNFa, IL6, CCL4, IL10, IL4, IL8, IL7, CCL16, IL1a, IL1b | Sweden | ALS(20)/DC(20) | 40.0/50.0 | 64.9/66.1 | NA | / | NA | multiplex PLA |
| Liu et al. 2015 | IFN-γ | China | ALS(52)/DC(31) | 69.2/61.3 | 52.0/49.4 | NA | / | El Escorial | ELISA |
| Mitchell et al. 2009 | IL-6, GM-CSF, IL-2, IL13,IL-15, IL-17, MIP1b, G-CSF, VEGF, MIP1a, MCP-1, IL-10, IFN-r , IL-4, IL-5, IL7, TNFa, IL-8, IL12(p70), IL1b, RANTES | America | ALS(41)/DC(33) | 65.9/33.3 | 58.8/43.9 | 16.6 | / | NA | Bio-Plex Human 27-plex panel/ ELISA |
| Moreau et al. 2005 | IL-6 | France | ALS(20)/DC(20) | 55.0/80.0 | NA | NA | / | El Escorial | ELISA |
| Nagata et al. 2007 | MCP-1,VEGF | Japan | ALS(42)/DC(25) /HC(16) | 71.4/44.0/  43.8 | 61.0/66.0/  72.0 | NA | / | El Escorial | ELISA |
| Rentzos et al. 2006 | IL-15 | Greece | ALS(19) /DC(7) | 31.6/55.6/28/42.9 | 61.0/56.0/64.0/58.0 | 1.8/2.7/2.7/2.0 | / | El Escorial | ELISA |
| Rentzos et al. 2007 | RANTES | Greece | ALS(20) /HC(13) | 65.0/71.4/61.5 | 56.0/58.0/52.0 | 13.0 | / | El Escorial | ELISA |
| Rentzos et al. 2010a | IL-17 | Greece | ALS(22)/DC(19) | 68.2/52.6 | 58.0/58.0 | 17.0 | / | El Escorial | ELISA |
| Rentzos et al. 2010b | IL-15 | Greece | ALS(21)/DC(19) | 66.7/52.6 | 56.0/58.0 | 17.0 | / | El Escorial | ELISA |
| Sekizawa et al. 1998 | IL-6 | Japan | ALS(27)/ DC(2) | NA | NA | NA | / | NA | NA |
| Tanaka et al. 2006 | IL-2, IL-4, IL-5, IL-6, IL-7, IL-8, IL-10, IL-12 (p70), IL-13, IL-17, IFN-γ, TNF-α, G-CSF, MCP-1, MIP-1β (CCL-4) | Japan | ALS(37)/DC(33) | 51.4/69.7 | 59.5/56.0 | 19.6 | / | El Escorial | Multiplexed Fluorescent Bead-Based immunoassay |
| Tateishi et al. 2010 | IL-2, IL-4, IL-5, IL-6, IL-10, IL-13, IL15,G-CSF, GM-CSF, VEGF, MCP-1, CCL5 (RANTES), CXCL8 (IL8), TNF-α, IFN-γ, IL-1β, IL-7, IL-12(p70), IL-17 | Japan | ALS(42)/ DC(34) | 47.6/50.0/61.8 | 56.7/55.2/54.2 | 13.0/25.9 | / | El Escorial | Multiplexed fluorescent bead-based immunoassay |
| Wilms et al. 2003 | MCP-1 | Germany | ALS(29)/DC(11) | NA | 61.6/56.8 | NA | / | El Escorial | ELISA |
| Yang et al. 2015 | MIP-1α | China | ALS(58)/DC(45) | 51.7/53.3 | 56.64/56.78 | NA | / | El Escorial | ELISA |
| Jie Guo et al. 2017 | IL-2, IL-6, IL-10, IL-15, IL-17, G-CSF, GM-CSF,VEGF, MIP-1α, MIP-1β, MCP-1, IFN-γ | China | ALS(105)/HC(56) | 53.3/55.4 | 58.0/56.98 | 30.7 | / | NA | ELISA |
| Drannik et al. 2017 | GM-CSF, IFN γ, IL-1β, IL-6, IL-8, IL-10, IL-17A, MCP-1, MIP1, TNF-α, VEGF | Canada | ALS(9)/HC(13) | 77.8/46.2 | 52.3/67.5 | 12.2 | / | NA | ELISA |

**Abbreviations:** AD, Alzheimer’s Disease; ALS, Amyotrophic lateral sclerosis; G-CSF, Granulocyte colony-stimulating factor; GM-CSF, Granulocyte-macrophage colony-stimulating factor; Ctrl, Control; HC, Healthy Control; DC, Disease Control H & Y, Hoehn and Yahr; IFN-γ, interferon γ; IL, interleukin; MCP, Monocyte Chemoattractant Protein; MIP, Macrophage Inflammatory Proteins; PD, Parkinson disease; RANTES, regulated on activation, normal T-expressed, and presumably secreted; TNF, tumor necrosis factor; TGF, transforming growth factor; VEGF, Vascular endothelial growth factor; YKL-40, Chitinase-3-like protein 1; ELISA, Enzyme-Linked ImmunoSorbent Assay; NINCDS/ADRDA, National Institute of Neurological and Communicative Diseases and Stroke/Alzheimer's Disease and Related Disorders Association; NA, not available;

**Supplementary References**：^1-71^

1. Alcolea D, Vilaplana E, Suarez-Calvet M, et al. CSF sAPPbeta, YKL-40, and neurofilament light in frontotemporal lobar degeneration. *Neurology.* 2017;89(2):178-188.

2. Antonell A, Mansilla A, Rami L, et al. Cerebrospinal fluid level of YKL-40 protein in preclinical and prodromal Alzheimer's disease. *Journal of Alzheimers Disease Jad.* 2014;42(3):901-908.

3. Baldacci F, Toschi N, Lista S, et al. Two-level diagnostic classification using cerebrospinal fluid YKL-40 in Alzheimer's disease. *Alzheimers & Dementia the Journal of the Alzheimers Association.* 2017;13(9).

4. Baron P, Bussini S, Cardin V, et al. Production of monocyte chemoattractant protein-1 in amyotrophic lateral sclerosis. *Muscle & nerve.* 2005;32(4):541-544.

5. Blasko I, Lederer W, Oberbauer H, et al. Measurement of thirteen biological markers in CSF of patients with Alzheimer's disease and other dementias. *Dementia and geriatric cognitive disorders.* 2006;21(1):9-15.

6. Blum-Degen D, Muller T, Kuhn W, Gerlach M, Przuntek H, Riederer P. Interleukin-1 beta and interleukin-6 are elevated in the cerebrospinal fluid of Alzheimer's and de novo Parkinson's disease patients. *Neuroscience letters.* 1995;202(1-2):17-20.

7. Correa JD, Starling D, Teixeira AL, Caramelli P, Silva TA. Chemokines in CSF of Alzheimer's disease patients. *Arquivos de neuro-psiquiatria.* 2011;69(3):455-459.

8. Delgado-Alvarado M, Gago B, Gorostidi A, et al. Tau/alpha-synuclein ratio and inflammatory proteins in Parkinson's disease: An exploratory study. *Movement disorders : official journal of the Movement Disorder Society.* 2017;32(7):1066-1073.

9. Drannik A, Martin J, Peterson R, Ma X, Jiang F, Turnbull J. Cerebrospinal fluid from patients with amyotrophic lateral sclerosis inhibits sonic hedgehog function. *PloS one.* 2017;12(2):e0171668.

10. Ford L, Rowe D. Interleukin-12 and interferon-gamma are not detectable in the cerebrospinal fluid of patients with amyotrophic lateral sclerosis. *Amyotrophic lateral sclerosis and other motor neuron disorders : official publication of the World Federation of Neurology, Research Group on Motor Neuron Diseases.* 2004;5(2):118-120.

11. Furukawa T, Matsui N, Fujita K, et al. CSF cytokine profile distinguishes multifocal motor neuropathy from progressive muscular atrophy. *Neurology(R) neuroimmunology & neuroinflammation.* 2015;2(5):e138.

12. Galimberti D, Schoonenboom N, Scheltens P, et al. Intrathecal chemokine synthesis in mild cognitive impairment and Alzheimer disease. *Archives of neurology.* 2006;63(4):538-543.

13. Galimberti D, Venturelli E, Fenoglio C, et al. Intrathecal levels of IL-6, IL-11 and LIF in Alzheimer's disease and frontotemporal lobar degeneration. *Journal of neurology.* 2008;255(4):539-544.

14. Gispert JD, Monte GC, Suarez-Calvet M, et al. The APOE epsilon4 genotype modulates CSF YKL-40 levels and their structural brain correlates in the continuum of Alzheimer's disease but not those of sTREM2. *Alzheimer's & dementia (Amsterdam, Netherlands).* 2017;6:50-59.

15. Gomez-Tortosa E, Gonzalo I, Fanjul S, et al. Cerebrospinal fluid markers in dementia with lewy bodies compared with Alzheimer disease. *Archives of neurology.* 2003;60(9):1218-1222.

16. Guo J, Yang X, Gao L, Zang D. Evaluating the levels of CSF and serum factors in ALS. *Brain and behavior.* 2017;7(3):e00637.

17. Gupta PK, Prabhakar S, Sharma S, Anand A. Vascular endothelial growth factor-A (VEGF-A) and chemokine ligand-2 (CCL2) in amyotrophic lateral sclerosis (ALS) patients. *Journal of neuroinflammation.* 2011;8:47.

18. Gupta PK, Prabhakar S, Sharma S, Anand A. A predictive model for amyotrophic lateral sclerosis (ALS) diagnosis. *Journal of the neurological sciences.* 2012;312(1-2):68-72.

19. Hampel H, Teipel SJ, Padberg F, et al. Discriminant power of combined cerebrospinal fluid tau protein and of the soluble interleukin-6 receptor complex in the diagnosis of Alzheimer's disease. *Brain research.* 1999;823(1-2):104-112.

20. Hu Y, Yu SY, Zuo LJ, et al. Parkinson disease with REM sleep behavior disorder: features, alpha-synuclein, and inflammation. *Neurology.* 2015;84(9):888-894.

21. Ilzecka J. Cerebrospinal fluid vascular endothelial growth factor in patients with amyotrophic lateral sclerosis. *Clinical neurology and neurosurgery.* 2004;106(4):289-293.

22. Janelidze S, Hertze J, Zetterberg H, et al. Cerebrospinal fluid neurogranin and YKL-40 as biomarkers of Alzheimer's disease. *Annals of clinical and translational neurology.* 2016;3(1):12-20.

23. Janelidze S, Lindqvist D, Francardo V, et al. Increased CSF biomarkers of angiogenesis in Parkinson disease. *Neurology.* 2015;85(21):1834-1842.

24. Krieger C, Perry TL, Ziltener HJ. Amyotrophic lateral sclerosis: interleukin-6 levels in cerebrospinal fluid. *The Canadian journal of neurological sciences. Le journal canadien des sciences neurologiques.* 1992;19(3):357-359.

25. Kuhle J, Lindberg RL, Regeniter A, et al. Increased levels of inflammatory chemokines in amyotrophic lateral sclerosis. *European journal of neurology.* 2009;16(6):771-774.

26. Lauridsen C, Sando SB, Moller I, et al. Cerebrospinal Fluid Abeta43 Is Reduced in Early-Onset Compared to Late-Onset Alzheimer's Disease, But Has Similar Diagnostic Accuracy to Abeta42. *Frontiers in aging neuroscience.* 2017;9:210.

27. Lee S, Tong M, Hang S, Deochand C, de la Monte S. CSF and Brain Indices of Insulin Resistance, Oxidative Stress and Neuro-Inflammation in Early versus Late Alzheimer's Disease. *Journal of Alzheimer's disease & Parkinsonism.* 2013;3:128.

28. Lehnert S, Costa J, de Carvalho M, et al. Multicentre quality control evaluation of different biomarker candidates for amyotrophic lateral sclerosis. *Amyotrophic lateral sclerosis & frontotemporal degeneration.* 2014;15(5-6):344-350.

29. Lind AL, Wu D, Freyhult E, et al. A Multiplex Protein Panel Applied to Cerebrospinal Fluid Reveals Three New Biomarker Candidates in ALS but None in Neuropathic Pain Patients. *PloS one.* 2016;11(2):e0149821.

30. Lindqvist D, Hall S, Surova Y, et al. Cerebrospinal fluid inflammatory markers in Parkinson's disease--associations with depression, fatigue, and cognitive impairment. *Brain, behavior, and immunity.* 2013;33:183-189.

31. Liu J, Gao L, Zang D. Elevated Levels of IFN-gamma in CSF and Serum of Patients with Amyotrophic Lateral Sclerosis. *PloS one.* 2015;10(9):e0136937.

32. Llano DA, Li J, Waring JF, et al. Cerebrospinal fluid cytokine dynamics differ between Alzheimer disease patients and elderly controls. *Alzheimer disease and associated disorders.* 2012;26(4):322-328.

33. Llorens F, Schmitz M, Knipper T, et al. Cerebrospinal Fluid Biomarkers of Alzheimer's Disease Show Different but Partially Overlapping Profile Compared to Vascular Dementia. *Frontiers in aging neuroscience.* 2017;9:289.

34. Maetzler W, Deleersnijder W, Hanssens V, et al. GDF15/MIC1 and MMP9 Cerebrospinal Fluid Levels in Parkinson's Disease and Lewy Body Dementia. *PloS one.* 2016;11(3):e0149349.

35. Martin de Pablos A, Garcia-Moreno JM, Fernandez E. Does the Cerebrospinal Fluid Reflect Altered Redox State But Not Neurotrophic Support Loss in Parkinson's Disease? *Antioxidants & redox signaling.* 2015;23(11):893-898.

36. Martinez M, Fernandez-Vivancos E, Frank A, De la Fuente M, Hernanz A. Increased cerebrospinal fluid fas (Apo-1) levels in Alzheimer's disease. Relationship with IL-6 concentrations. *Brain research.* 2000;869(1-2):216-219.

37. Mattsson N, Tabatabaei S, Johansson P, et al. Cerebrospinal fluid microglial markers in Alzheimer's disease: elevated chitotriosidase activity but lack of diagnostic utility. *Neuromolecular medicine.* 2011;13(2):151-159.

38. Mitchell RM, Freeman WM, Randazzo WT, et al. A CSF biomarker panel for identification of patients with amyotrophic lateral sclerosis. *Neurology.* 2009;72(1):14-19.

39. Mogi M, Harada M, Narabayashi H, Inagaki H, Minami M, Nagatsu T. Interleukin (IL)-1 beta, IL-2, IL-4, IL-6 and transforming growth factor-alpha levels are elevated in ventricular cerebrospinal fluid in juvenile parkinsonism and Parkinson's disease. *Neuroscience letters.* 1996;211(1):13-16.

40. Mogi M, Harada M, Riederer P, Narabayashi H, Fujita K, Nagatsu T. Tumor necrosis factor-alpha (TNF-alpha) increases both in the brain and in the cerebrospinal fluid from parkinsonian patients. *Neuroscience letters.* 1994;165(1-2):208-210.

41. Moreau C, Devos D, Brunaud-Danel V, et al. Elevated IL-6 and TNF-alpha levels in patients with ALS: inflammation or hypoxia? *Neurology.* 2005;65(12):1958-1960.

42. Muller T, Blum-Degen D, Przuntek H, Kuhn W. Interleukin-6 levels in cerebrospinal fluid inversely correlate to severity of Parkinson's disease. *Acta neurologica Scandinavica.* 1998;98(2):142-144.

43. Nagata T, Nagano I, Shiote M, et al. Elevation of MCP-1 and MCP-1/VEGF ratio in cerebrospinal fluid of amyotrophic lateral sclerosis patients. *Neurological research.* 2007;29(8):772-776.

44. Olsson B, Hertze J, Lautner R, et al. Microglial markers are elevated in the prodromal phase of Alzheimer's disease and vascular dementia. *Journal of Alzheimer's disease : JAD.* 2013;33(1):45-53.

45. Paterson RW, Slattery CF, Poole T, et al. Cerebrospinal fluid in the differential diagnosis of Alzheimer's disease: clinical utility of an extended panel of biomarkers in a specialist cognitive clinic. *Alzheimer's research & therapy.* 2018;10(1):32.

46. Paterson RW, Toombs J, Slattery CF, et al. Dissecting IWG-2 typical and atypical Alzheimer's disease: insights from cerebrospinal fluid analysis. *Journal of neurology.* 2015;262(12):2722-2730.

47. Pirttila T, Mehta PD, Frey H, Wisniewski HM. Alpha 1-antichymotrypsin and IL-1 beta are not increased in CSF or serum in Alzheimer's disease. *Neurobiology of aging.* 1994;15(3):313-317.

48. Portelius E, Soininen H, Andreasson U, et al. Exploring Alzheimer molecular pathology in Down's syndrome cerebrospinal fluid. *Neuro-degenerative diseases.* 2014;14(2):98-106.

49. Rentzos M, Nikolaou C, Rombos A, et al. RANTES levels are elevated in serum and cerebrospinal fluid in patients with amyotrophic lateral sclerosis. *Amyotrophic lateral sclerosis : official publication of the World Federation of Neurology Research Group on Motor Neuron Diseases.* 2007;8(5):283-287.

50. Rentzos M, Rombos A, Nikolaou C, et al. Interleukin-15 and interleukin-12 are elevated in serum and cerebrospinal fluid of patients with amyotrophic lateral sclerosis. *European neurology.* 2010;63(5):285-290.

51. Rentzos M, Rombos A, Nikolaou C, et al. Interleukin-17 and interleukin-23 are elevated in serum and cerebrospinal fluid of patients with ALS: a reflection of Th17 cells activation? *Acta neurologica Scandinavica.* 2010;122(6):425-429.

52. Rentzos M, Zoga M, Paraskevas GP, et al. IL-15 is elevated in cerebrospinal fluid of patients with Alzheimer's disease and frontotemporal dementia. *Journal of geriatric psychiatry and neurology.* 2006;19(2):114-117.

53. Richartz E, Stransky E, Batra A, et al. Decline of immune responsiveness: a pathogenetic factor in Alzheimer's disease? *Journal of psychiatric research.* 2005;39(5):535-543.

54. Rosen C, Andersson CH, Andreasson U, et al. Increased Levels of Chitotriosidase and YKL-40 in Cerebrospinal Fluid from Patients with Alzheimer's Disease. *Dementia and geriatric cognitive disorders extra.* 2014;4(2):297-304.

55. Rosler N, Wichart I, Jellinger KA. Intra vitam lumbar and post mortem ventricular cerebrospinal fluid immunoreactive interleukin-6 in Alzheimer's disease patients. *Acta neurologica Scandinavica.* 2001;103(2):126-130.

56. Rota E, Bellone G, Rocca P, Bergamasco B, Emanuelli G, Ferrero P. Increased intrathecal TGF-beta1, but not IL-12, IFN-gamma and IL-10 levels in Alzheimer's disease patients. *Neurological sciences : official journal of the Italian Neurological Society and of the Italian Society of Clinical Neurophysiology.* 2006;27(1):33-39.

57. Sekizawa T, Openshaw H, Ohbo K, Sugamura K, Itoyama Y, Niland JC. Cerebrospinal fluid interleukin 6 in amyotrophic lateral sclerosis: immunological parameter and comparison with inflammatory and non-inflammatory central nervous system diseases. *Journal of the neurological sciences.* 1998;154(2):194-199.

58. Tanaka M, Kikuchi H, Ishizu T, et al. Intrathecal upregulation of granulocyte colony stimulating factor and its neuroprotective actions on motor neurons in amyotrophic lateral sclerosis. *Journal of neuropathology and experimental neurology.* 2006;65(8):816-825.

59. Tarkowski E, Blennow K, Wallin A, Tarkowski A. Intracerebral production of tumor necrosis factor-alpha, a local neuroprotective agent, in Alzheimer disease and vascular dementia. *Journal of clinical immunology.* 1999;19(4):223-230.

60. Tarkowski E, Issa R, Sjogren M, et al. Increased intrathecal levels of the angiogenic factors VEGF and TGF-beta in Alzheimer's disease and vascular dementia. *Neurobiology of aging.* 2002;23(2):237-243.

61. Tarkowski E, Liljeroth AM, Nilsson A, et al. TNF gene polymorphism and its relation to intracerebral production of TNFalpha and TNFbeta in AD. *Neurology.* 2000;54(11):2077-2081.

62. Tarkowski E, Wallin A, Regland B, Blennow K, Tarkowski A. Local and systemic GM-CSF increase in Alzheimer's disease and vascular dementia. *Acta neurologica Scandinavica.* 2001;103(3):166-174.

63. Tateishi T, Yamasaki R, Tanaka M, et al. CSF chemokine alterations related to the clinical course of amyotrophic lateral sclerosis. *Journal of neuroimmunology.* 2010;222(1-2):76-81.

64. Vawter MP, Dillon-Carter O, Tourtellotte WW, Carvey P, Freed WJ. TGFbeta1 and TGFbeta2 concentrations are elevated in Parkinson's disease in ventricular cerebrospinal fluid. *Experimental neurology.* 1996;142(2):313-322.

65. Wennstrom M, Hall S, Nagga K, Londos E, Minthon L, Hansson O. Cerebrospinal fluid levels of IL-6 are decreased and correlate with cognitive status in DLB patients. *Alzheimer's research & therapy.* 2015;7(1):63.

66. Wilms H, Sievers J, Dengler R, Bufler J, Deuschl G, Lucius R. Intrathecal synthesis of monocyte chemoattractant protein-1 (MCP-1) in amyotrophic lateral sclerosis: further evidence for microglial activation in neurodegeneration. *Journal of neuroimmunology.* 2003;144(1-2):139-142.

67. Yamada K, Kono K, Umegaki H, et al. Decreased interleukin-6 level in the cerebrospinal fluid of patients with Alzheimer-type dementia. *Neuroscience letters.* 1995;186(2-3):219-221.

68. Yang X, Gao L, Wu X, Zhang Y, Zang D. Increased levels of MIP-1alpha in CSF and serum of ALS. *Acta neurologica Scandinavica.* 2016;134(2):94-100.

69. Yu SY, Zuo LJ, Wang F, et al. Potential biomarkers relating pathological proteins, neuroinflammatory factors and free radicals in PD patients with cognitive impairment: a cross-sectional study. *BMC neurology.* 2014;14:113.

70. Zetterberg H, Andreasen N, Blennow K. Increased cerebrospinal fluid levels of transforming growth factor-beta1 in Alzheimer's disease. *Neuroscience letters.* 2004;367(2):194-196.

71. Zhang J, Sokal I, Peskind ER, et al. CSF multianalyte profile distinguishes Alzheimer and Parkinson diseases. *American journal of clinical pathology.* 2008;129(4):526-529.
